# Microencapsulated essential oils alleviate diarrhea in weaned piglets by modulating the intestinal microbial barrier as well as not inducing antibiotic resistance: a field research

**DOI:** 10.3389/fvets.2024.1396051

**Published:** 2024-05-10

**Authors:** Xianbin Xu, Kaibin Mo, Can Cui, Yanhua Lan, Lifang Ling, Jinxia Xu, Li Li, Xianhui Huang

**Affiliations:** ^1^Guangdong Key Laboratory for Veterinary Drug Development and Safety Evaluation, College of Veterinary Medicine, South China Agricultural University, Guangzhou, China; ^2^College of Animal Science, South China Agricultural University, Guangzhou, China

**Keywords:** microencapsulated essential oils, piglet diarrhea, intestinal health, metagenomic, metabolomic

## Abstract

Microencapsulated essential oils (MEO)have been used as antibiotic alternatives that can be applied to alleviate diarrhea in weaning piglet. We examined a large group of weaned piglets and incorporated essential oil containing thymol (2%), carvacrol (5%) and cinnamaldehyde (3%) in the feed of weaned piglets on an intensive production farm. The piglets were divided into four groups; Control (no additions) and chlortetracycline (Chl), essential oil (EO) and microencapsulated essential oil (MEO) were fed *ad libitum* over a 28-day trial period. We found MEO significantly reduced the incidence of diarrhea in the piglets that was also accompanied by increased average daily weight gains from days 14–28 (*p* < 0.05). MEO enhanced the antioxidant capacity in the piglets and serum total antioxidant capacity (T-AOC) and glutathione peroxidase (GSH-px) levels were significantly increased (*p* < 0.05). MEO also significantly reduced expression of genes related to ileal inflammation (*IL-6*, *TNF-α* and *TGF-β1*) (*p* < 0.05) and significantly (*p* < 0.05) increased in sIgA antibody levels. MEO influenced the composition of the intestinal microbiome and reduced Bacteroidota (*p* < 0.05) and thus altered the Firmicutes/Bacteroidota ratio. However, none of the treatments produced significant changes in the most common tetracycline resistance genes (*p* > 0.05). Metagenomic analysis indicated that MEO impacted DNA expression, virulence factors, antioxidant activity and antimicrobial activity. Metabolomic analysis of the intestinal content also indicated that MEO impacted tyrosine metabolism and primary bile acid biosynthesis suggesting improved intestinal health and nutrient absorption. This study paves the way for further research into the development and optimization of MEO-based interventions aimed at improving piglet health and performance while also providing a reference for reducing reliance on antibiotics in animal agriculture.

## 1 Introduction

Weaning diarrhea found in piglets is a common and significant economic issue in agricultural production since these animals exhibit slow growth, and both decreased feed efficiency and survival (1). The abrupt diet alterations necessary for weaning coupled with an immature intestine result in disorder of the intestinal microbiota leading to damage of intestinal barriers that triggers diarrhea (2). At a microscopic level, weaned piglets display insufficient secretion of digestive enzymes in the gastrointestinal tract and this hinders solid food digestion (3). This process also damages cellular tight junctions (4), reduces mucin production (5) that both lead to increases intestinal permeability (6). These processes also alter the intestinal microbiota (7) that can allow for invasion by pathogenic microbes (8) that can set up an inflammatory process (9) that further exacerbates barrier dysfunction at the intestinal mucosa.

The modern era of restricted or even prohibited use of antibiotics in feed supplements has necessitated the search for alternatives to maintain animal intestinal health (10). Plant essential oils including natural bioactive compounds have exhibited antimicrobial, anti-inflammatory antioxidant even anti-tumor activities (11). The inclusion of plant essential oils and organic acids into the weaning diet can decrease intestinal inflammation and reduce diarrhea, positively impact cellular and humoral immunity, improve intestinal morphology and antioxidant capacity of the ileal mucosa leading to a balanced cecal microbiota (12–14). In particular, experiments that involve addition of thymol, cinnamaldehyde and enzyme mixtures to piglet diets decreased expression of ileal inflammatory biomarkers and decreased diathermal incidence (15). However, these types of essential oils are unstable and procedures such as microencapsulation are necessary to ensure activity is maintained during transit through the gastrointestinal tract and provides the added benefit of enhancing palatability (16). Microencapsulated essential oils given to weaning piglets can significantly alleviate intestinal oxidative stress and inflammation and overall positively affect growth performance and intestinal integrity (17).

Previous studies have demonstrated that microencapsulated essential oils (MEO) consisting of carvacrol, thymol and cinnamaldehyde reduce the occurrence of diarrhea after weaning and promote growth performance. These benefits were observed utilizing piglets fed individually in separate experimental replicates and this protocol represented an incomplete growth environment (18). The current study was performed to expand the results of that study using increased sample sizes and explored MEO influences on piglet intestinal barrier function using a field research. Additionally, our study endeavored to determine whether MEO can alter the microbiome and whether this is accompanied by changes in antibiotic resistance of intestinal bacteria.

## 2 Materials and methods

### 2.1 Dietary supplement preparation

Chlortetracycline (Chl), normal essential oils (EO) and microencapsulated essential oils (MEO) were all provided by Jinhe Biotechnology, China. Chlortetracycline was in granular form (10%) and EO and MEO equally contained 2% thymol, 5% carvacrol and 3% cinnamaldehyde.

### 2.2 Animals, grouping and treatment

A total of 288 weaned piglets with consistent health status and genetic background, half male and half female and were randomly divided into four groups and fed identical NRC standard pig farm formulation (19) modified as follows: Control no additions; Chl, EO and MEO added at 100 mg/kg each. There were 6 replicates per group and each replicate housed 12 piglets in one pen (4 × 3 m). The experiment lasted for 28 days and was conducted on an intensive farm (Guanghui Agriculture and Animal Husbandry, China). During the experiment, the piglets had free access to water and feed and the temperature inside the pens were maintained at 27 ± 3°C.

Piglet weights and feed consumption was measured on days 1, 14 and 28 of the experiment. The presence of diarrhea, average daily gain (ADG), average daily feed intake (ADFI) and the feed/gain ratio (F/G) were calculated as previously described study (20).

### 2.3 Sample collection

On days 14 and 28, two healthy piglets with body weights close to the average were selected from each replicate (12 per group) and 5 mL blood was collected and serum was obtained after centrifugation and stored at −20°C until analysis. Immediately following the last blood collection on day 28, two piglets per replicate were euthanized. The duodenum, middle jejunum and middle ileum were stored in 4% formaldehyde while the jejunum mucosa, ileum mucosa and colonic content were snap-frozen in liquid nitrogen and stored at −80°C for further analysis as previously described (18). Additionally, we collected samples from the colonic contents for bacterial culture using sterile swabs, and preserved in 4°C. The culture process was performed within 18 h after the samples were collected.

### 2.4 Serum biomarkers analysis

Total antioxidant capacity (T-AOC), and glutathione peroxidase (GSH-Px) activities in serum were determined with commercial reagent kits (Nanjing Jiancheng Bioengineering Institute, China) and Microplate reader (PerkinElmer, United States).

### 2.5 Intestinal morphology analysis

Hematoxylin and eosin (H&E) stained slides were made as previously described (18) and observed and photographed using a slide scanning system (3D Histech, Hungary). For analysis, 10 villi and crypts were randomly selected in each photo to measure villus height (VH) and crypt depth (CD), and to calculate the villus height to crypt depth ratio (VH/CD).

### 2.6 Intestinal barrier biomarkers analysis

Target gene biomarkers in the jejunum and ileum mucosa included *MUC2*, *occludin*, *claudin-1*, zonula occludens tight junction protein-1 (*ZO-1*), interleukins (IL) *IL-1β*, *IL-6* and *IL-10*, tumor necrosis factor α (*TNF-α*), interferon*-γ* (*IFN-γ*) and transforming growth factor (*TGF-β1*). The CT scores for these genes were normalized to the internal reference gene *GAPDH* (Supplementary Table S1). RNA extraction of homogenized tissues and reverse transcription and qualification methods were performed as previously described (18).

Total protein, secretory immunoglobulin A (sIgA) and intestinal trefoil factor (ITF) levels in the jejunum and ileum mucosa were quantified using a commercial BCA reagent kit (Sangon Biotech, China) and ELISA kits (Jiangsu Meimian Industry, China). The data were normalized to the total protein concentration during analysis.

### 2.7 Analysis of tetracycline resistance genes from intestinal *Escherichia coli*

Colon contents samples (1 g) were cultured in Luria-Bertani (LB) broth at 37°C for 18 h then plated on MacConkey agar. *Escherichia coli* were identified as red colonies that were then cultured in LB broth for another 18 h. Bacterial DNA was extracted using a commercial Total DNA extraction kit (TransGen Biotech, China). DNA amplification was performed using a commercial qPCR reagent kit (Vazyme Biotech, China) in CFX96 Real-Time PCR Detection System (Bio-Rad Laboratories, United States) to determine DNA copy levels of the Tet-resistance genes *tetA, tetB*, *tetC*, *tetM*, *tetO* and *tetW*. Data were normalized to 16S rDNA (Supplementary Table S2). Gene amplicon quality was assessed vis melting curve analysis to ensure single amplicon (Supplementary Figure S8).

### 2.8 Intestinal DNA analysis

Metagenome analysis of microbiome DNA was carried out using 6 random samples per group and genomic DNA was extracted using the DNeasy PowerSoil Kit (Qiagen, Netherlands) and assessed for quality using agarose gel electrophoresis of 20–60 ng samples (Supplementary Figure S1).

High-throughput metagenomic sequencing was conducted on the Illumina platform (Illumina, United States) by Personal Biotech, China using a PE150 whole-genome shotgun strategy that resulted in high-quality reads with host contamination removed. Gene prediction and annotation were conducted using the GTDB database as previously described (21, 22). All annotated genes expressed as transcripts per kilobase per million mapped reads (TPM) that indicated the proportion of a particular gene present in the total gene abundance multiplied by a million. Gene functions were annotated using databases including KEGG, eggNOG, GO, CAZy, CARD, Metacyc, MCycDB, PCycDB, NCycDB, SCycDB, Pfam, PHI, VFDB, Swiss-Prot.

Alpha-diversity and beta-diversity based on Bray–Curtis distances as well as species differential analysis were performed using QIIME II software (23) that included principal component analysis (PCA). Venn diagrams and LEfSe (LDA Effect Size) differential analysis were generated with R software 4.33 (24).

### 2.9 Intestinal metabolite extraction and analysis

Metabolites in the colonic contents were extracted with methanol and quantified using an ultra-high performance liquid chromatography/mass spectrometry (UHPLC-MS) system (25) on a Thermo Vanquish (Thermo Fisher Scientific, United States) as previously detailed (26) and a Thermo Orbitrap Exploris 120 mass spectrometer (Thermo Fisher) using data collection from both positive and negative ion modes as previously described (27).

PCA and orthogonal partial least squares discriminant analysis (OPLS-DA) were conducted in R software to analyze and screen metabolites. Potential biomarkers were selected based on VIP scores >1 in OPLS-DA. Simultaneously, metabolites were screened further and those with FC >2 and *p*-value <0.05 in student’s *t*-test were selected.

Differential metabolites from optimized data underwent substance enrichment analysis in R software with reference to the KEGG database to determine host pig metabolic pathways.

### 2.10 Statistical analysis

All data not were computed and analyzed using Excel (Microsoft, United States) and SPSS software 25.0 (IBM, United States). Differences in multi-group data were analyzed using one-way ANOVA and between two groups using student’s *t*-test. A *p*-value <0.05 was considered statistically significant. The bar graphs were generated by Prism 8 (GraphPad, United States). PCR gene expression values were normalized using the 2^−ΔΔCt^ method.

## 3 Results

### 3.1 Growth performance

Our experimental diets containing MEO significantly (*p* < 0.05) reduced the incidence of diarrhea for days 1–14 when compared to the Control group. In addition, MEO increased the average daily gain (ADG) for days 15–28 (*p* < 0.05) and the Feed/Gain ratio (F/G) across the entire growth period (days 1–28) (*p* < 0.05) (Table 1).

**Table 1:** Effects of MEO supplementation on the grow performance.

	Control	Chl	EO	MEO	*p*-value
**Days 1–14**					
Diarrhea incidence	0.07 ± 0.01^ab^	0.04 ± 0.01^bc^	0.06 ± 0.01^ab^	0.04 ± 0.01^c^	0.026
ADFI	164.11 ± 1.62	164.37 ± 0.42	154.50 ± 4.99	168.85 ± 5.72	0.147
ADG	45.08 ± 5.92	51.59 ± 3.95	42.66 ± 2.50	59.03 ± 3.54	0.081
F/G	4.02 ± 0.50	3.31 ± 0.27	3.68 ± 0.21	2.89 ± 0.09	0.131
**Days 15–28**					
Diarrhea incidence	0.06 ± 0.02	0.02 ± 0.01	0.05 ± 0.01	0.01 ± 0.00	0.112
ADFI	352.86 ± 11.95	355.22 ± 6.54	362.22 ± 10.73	388.37 ± 12.05	0.148
ADG	143.21 ± 10.91^a^	154.37 ± 6.55^a^	160.83 ± 6.95^ab^	189.29 ± 11.14^b^	0.025
F/G	2.54 ± 0.18	2.32 ± 0.07	2.27 ± 0.07	2.08 ± 0.08	0.089
**Days 1–28**					
Diarrhea incidence	0.06 ± 0.01^ab^	0.03 ± 0.01^bc^	0.06 ± 0.01^ab^	0.02 ± 0.01^bc^	0.027
ADFI	258.48 ± 6.32	259.79 ± 3.19	258.36 ± 6.21	278.61 ± 8.42	0.140
ADG	94.15 ± 7.29	102.98 ± 4.28	101.75 ± 4.20	124.16 ± 6.73	0.081
F/G	2.82 ± 0.17^a^	2.54 ± 0.09^ab^	2.56 ± 0.08^ab^	2.27 ± 0.07^b^	0.034

Data are represented as means ± SEM, *n* = 6 per group. ^a,b,c^Values with different letters were significantly different (*p* < 0.05).

### 3.2 Serum antioxidant levels

**Figure 1:**
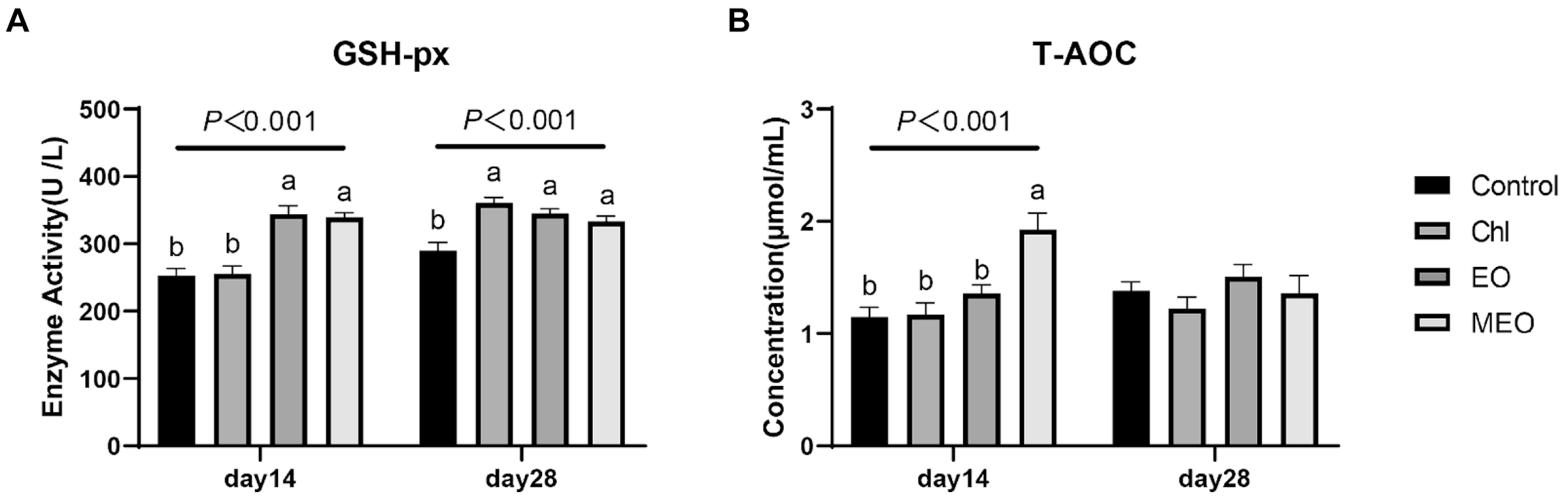
Effects of MEO supplementation on the serum antioxidant and immune biomarkers. **(A)** GSH-px **(B)** T-AOC. Data are represented as means ± SEM, *n* = 12 per group. ^a,b^Values with different letters were significantly different (*p* < 0.05). Diets are listed in the Materials and methods section.

The MEO and EO groups also displayed significantly increased levels of serum GSH-px (day 14, *p* < 0.001; day 28, *p* < 0.001) (Figure 1A). This was also positively correlated with the T-AOC in the MEO group that increased significantly (day 14, *p* < 0.001) (Figure 1B).

### 3.3 Intestinal physical and chemical barrier biomarkers

**Figure 2:**
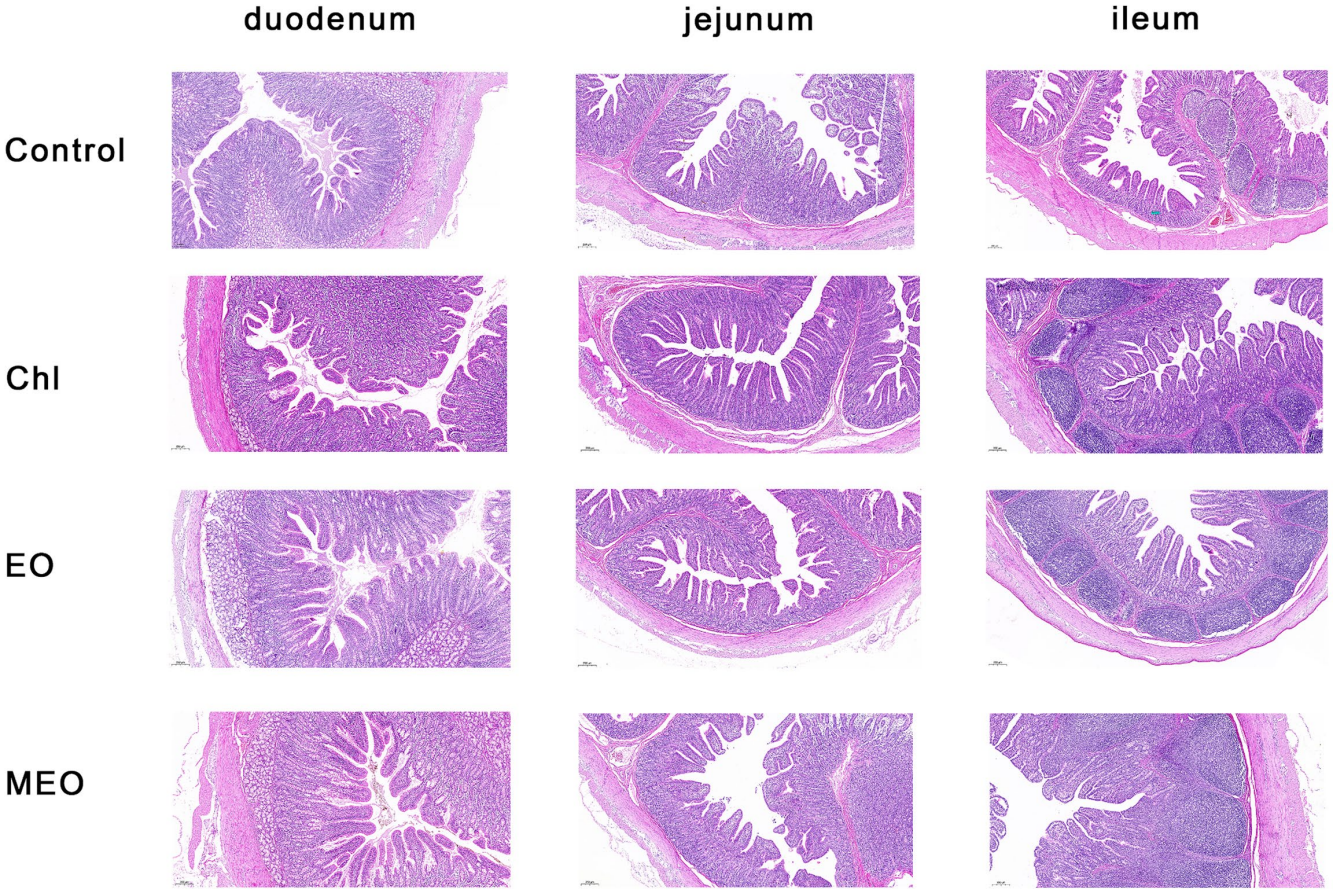
Effects of MEO supplementation on the intestinal morphology in weaning piglets. H&E staining of mucosal sections from the duodenum, jejunum, and ileum as indicated (magnification 5×).

We also examined histological sections of the duodenum, jejunum and ileum from our experimental animals. We could find no visible damage for either the control or experimental groups (Figure 2). Interestingly, jejunal villus height was significantly (*p* = 0.003) increased in the MEO and EO groups compared with the control (Table 2).

**Table 2:** Effects of MEO supplementation on the intestinal epithelial tissue.

	Control	Chl	EO	MEO	*p*-value
**VH (μm)** ^†^					
Duodenum	322.69 ± 6.88	339.93 ± 19.17	349.33 ± 22.08	350.52 ± 16.18	0.704
Jejunum	289.69 ± 8.21^a^	292.00 ± 14.57^a^	334.35 ± 9.15^b^	344.27 ± 6.25^b^	0.003
Ileum	294.79 ± 21.89	314.05 ± 13.55	301.63 ± 9.18	316.75 ± 10.86	0.744
**CD (μm)** ^‡^					
Duodenum	260.87 ± 7.62	297.41 ± 6.72	317.71 ± 21.53	305.26 ± 12.84	0.072
Jejunum	216.60 ± 4.98	227.85 ± 11.81	234.95 ± 5.52	234.15 ± 9.81	0.497
Ileum	218.11 ± 6.46	233.82 ± 9.31	235.66 ± 8.63	233.05 ± 9.36	0.533
VH/CD					
Duodenum	1.24 ± 0.03	1.14 ± 0.06	1.11 ± 0.06	1.15 ± 0.05	0.408
Jejunum	1.34 ± 0.05^a^	1.28 ± 0.02^ab^	1.42 ± 0.03^ab^	1.49 ± 0.07^b^	0.045
Ileum	1.35 ± 0.09	1.35 ± 0.05	1.29 ± 0.05	1.37 ± 0.05	0.861

^†^Vili height. ^‡^Crypt depth. Data are represented as means ± SEM, *n* = 6 per group. ^a,b,c^Values with different letters were significantly different (*p* < 0.05).

**Figure 3:**
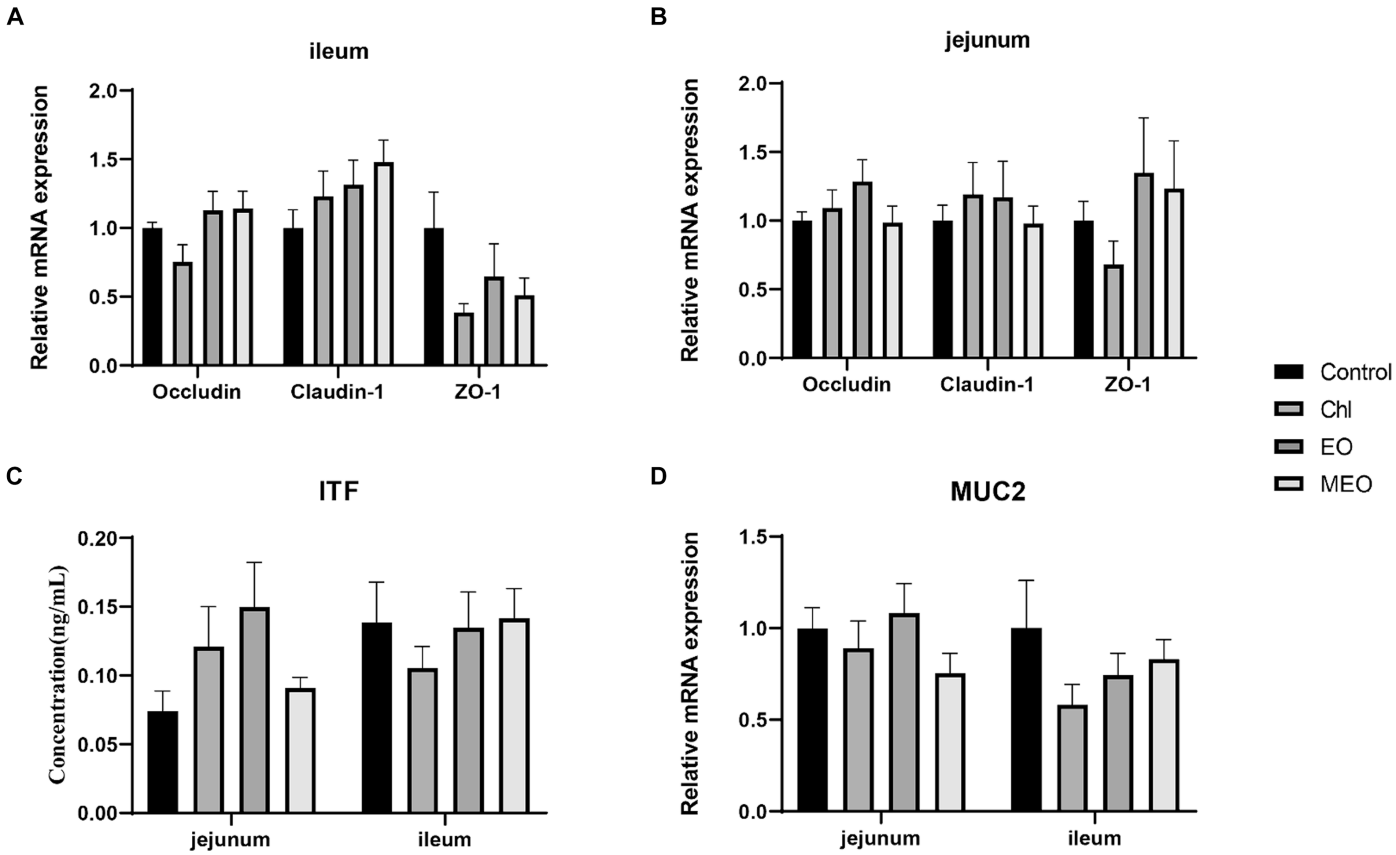
Effects of MEO supplementation on the intestinal physical barrier biomarkers. Relative expression levels of *OCCN*, *CLDN-1* and *ZO-1* in the **(A)** ileum and **(B)** jejunum **(C)** ITF protein levels in jejunal and ileal tissues **(D)** Relative expression of *MUC* in the ileum and jejunum. Data are represented as means ± SEM, *n* = 12 per group.

However, the MEO did not significantly (*p* > 0.05) alter intestinal physical barrier indicators of the intestine (Figures 3A,B). In particular, *MUC2* expression levels and ITF protein concentrations between the control and other treatment groups were not significantly (*p* > 0.05) different (Figures 3C,D).

### 3.4 Intestinal immune barrier biomarkers

**Figure 4:**
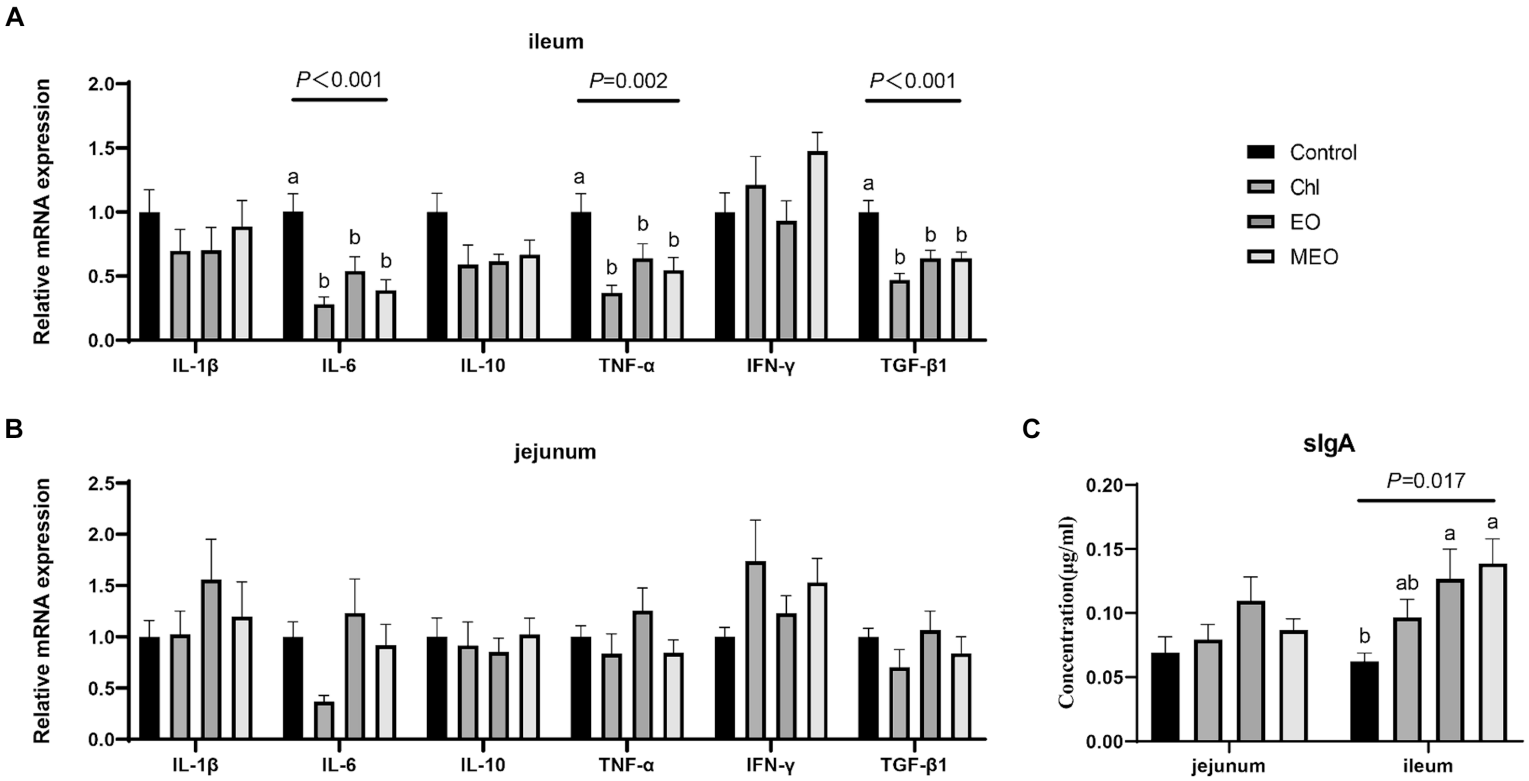
Effects of MEO supplementation on the intestinal immune barrier biomarkers. Relative mRNA expression levels of *IL-1β*, *IL-6*, *IL-10*, *TNF-α*, *IFN-γ* and *TGF-β1* in the **(A)** ileum and **(B)** jejunum. **(C)** sIgA protein levels in jejunal and ileal tissue. Data are represented as means ± SEM, *n* = 12 per group. ^a,b^Values with different letters were significantly different (*p* < 0.05).

We also examined the expression of intestinal inflammatory biomarkers in our experimental animals to assess whether our treatments could alter intestinal inflammation. Compared to the Control group, the MEO, EO and Chl group displayed significantly reduced expression of inflammatory biomarkers *IL-6* (*p* < 0.05), *TNF-α* (*p* < 0.05) and *TGF-β1* (*p* < 0.05) in the ileum of weaned piglets (Figure 4A). However, there were no significant difference observed in jejunum (Figure 4B). Additionally, the MEO and EO groups displayed significant (*p* = 0.017) increases in the levels of sIgA protein in the ileum (Figure 4C).

### 3.5 Intestinal microbial barriers

#### 3.5.1 Intestinal microbial species

**Figure 5:**
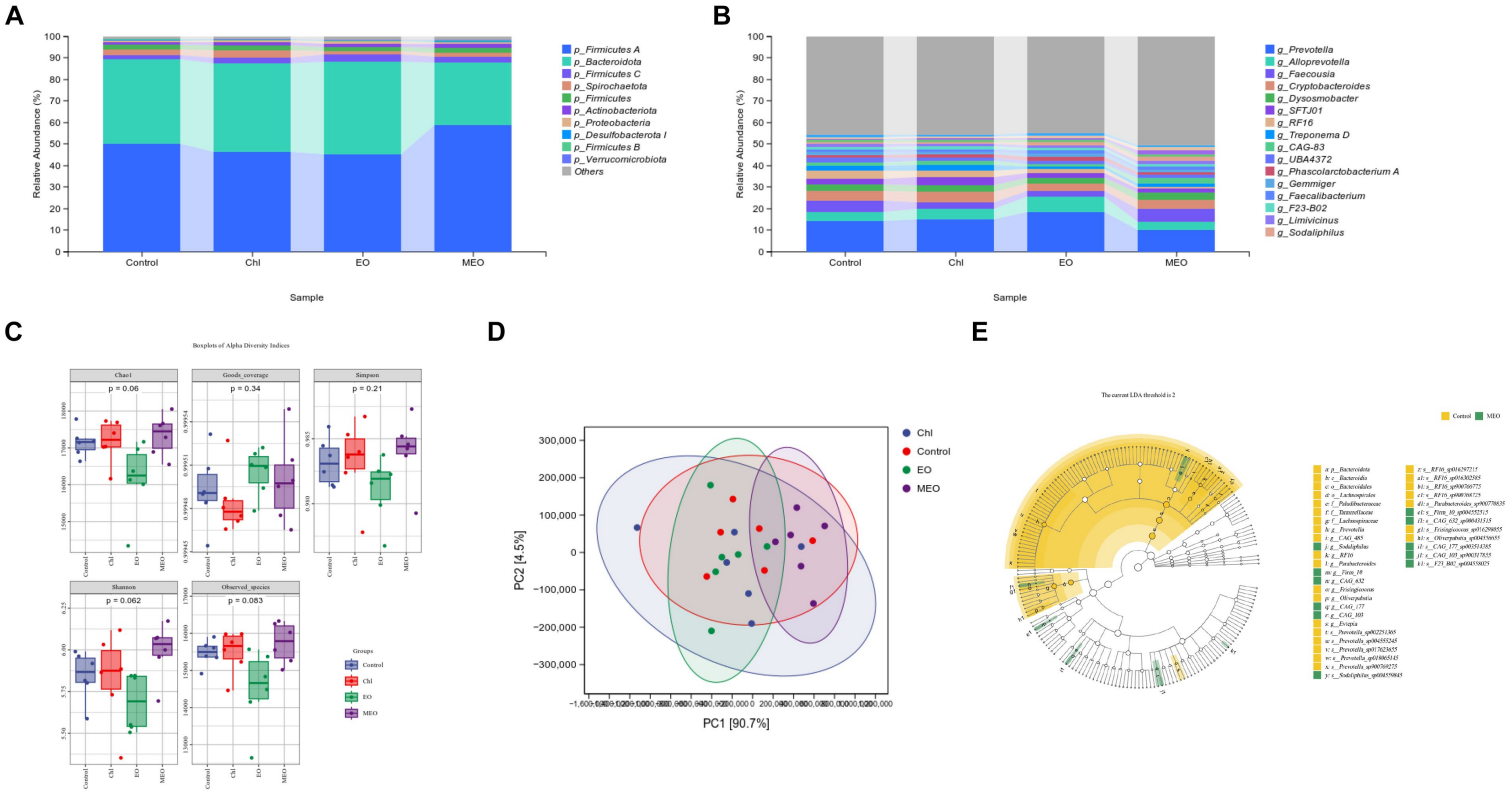
Effects of MEO supplementation on the intestinal microbial species. **(A)** Average relative abundance of colonic microbiota at the phylum level. **(B)** Average relative abundance of colonic microbiota at the genus level. **(C)** α-diversity indices among experimental groups. **(D)** PCA analysis at the phylum level of colonic microbiota. **(E)** LEfSe taxonomic cladogram depicting scores contributing to species differences and the relationship of differential species from the phylum to the genus level.

The microbial species composition of the colonic content indicated that the phyla *Firmicutes* A and *Bacteroidetes* dominated accounting for 86.67–89.53% of the total abundance. The 10 most abundant phyla in terms of relative abundance had a similar proportion structure among all groups. For instance, compared to the controls, the MEO group had an 8.59% increase in the average abundance of *Firmicutes* A and a 10.17% decrease in the average abundance of *Bacteroidota* that increased the *Firmicutes* / *Bacteroidota* (F/B) ratio Pathogenic phyla including *Spirochaetota*, *Actinobacteriota*, and *Proteobacteria* accounted for <5% of the total abundance for all four groups. Interestingly, *Bacteroidota* abundance significantly decreased in the MEO group and indicated that MEO directly influenced the intestinal microbiota. In contrast, this effect was not present in the EO and Chl groups (Figure 5A). In addition, the MEO group displayed a significant decrease in *Prevotella* and a significant (*p* < 0.05) increase in *Sodaliphilus* (both phylum Bacteroidota) and *CAG-83* and *CAG-103* genera (phylum Firmicutes A). Also, we did not observe and differential genera in the EO and Chl groups (Figure 5B).

#### 3.5.2 Intestinal microbial α- and β-diversity

Our experimental groups were also analyzed for α-diversity and no significant differences between groups were found (Figure 5C). PCA analysis at the phylum level revealed differences between the control and MEO groups (Figure 5D). The LEfSe cladogram also indicated the presence of differential species between the control and MEO groups (Figure 5E). In particular, some of these taxons were significantly reduced in the MEO group and included 10 species across 4 genera in the under Bacteroidota phylum; *Prevotella*, *Parabacteroides*, *RF16* and *CAG 485* along with two species across three genera in the *Firmicutes* A phylum; *Evtepia*, *Frisingicoccus* and *Oliverpabstia*. Species that were significantly increased included one species of *Sodaliphilus* genus (Bacteroidota) and 5e species across four genera (*Firmicutes* A); *Firm 10*, *CAG-632*, *CAG-177* and *CAG-103*.

#### 3.5.3 Intestinal microbial functional genes

**Figure 6:**
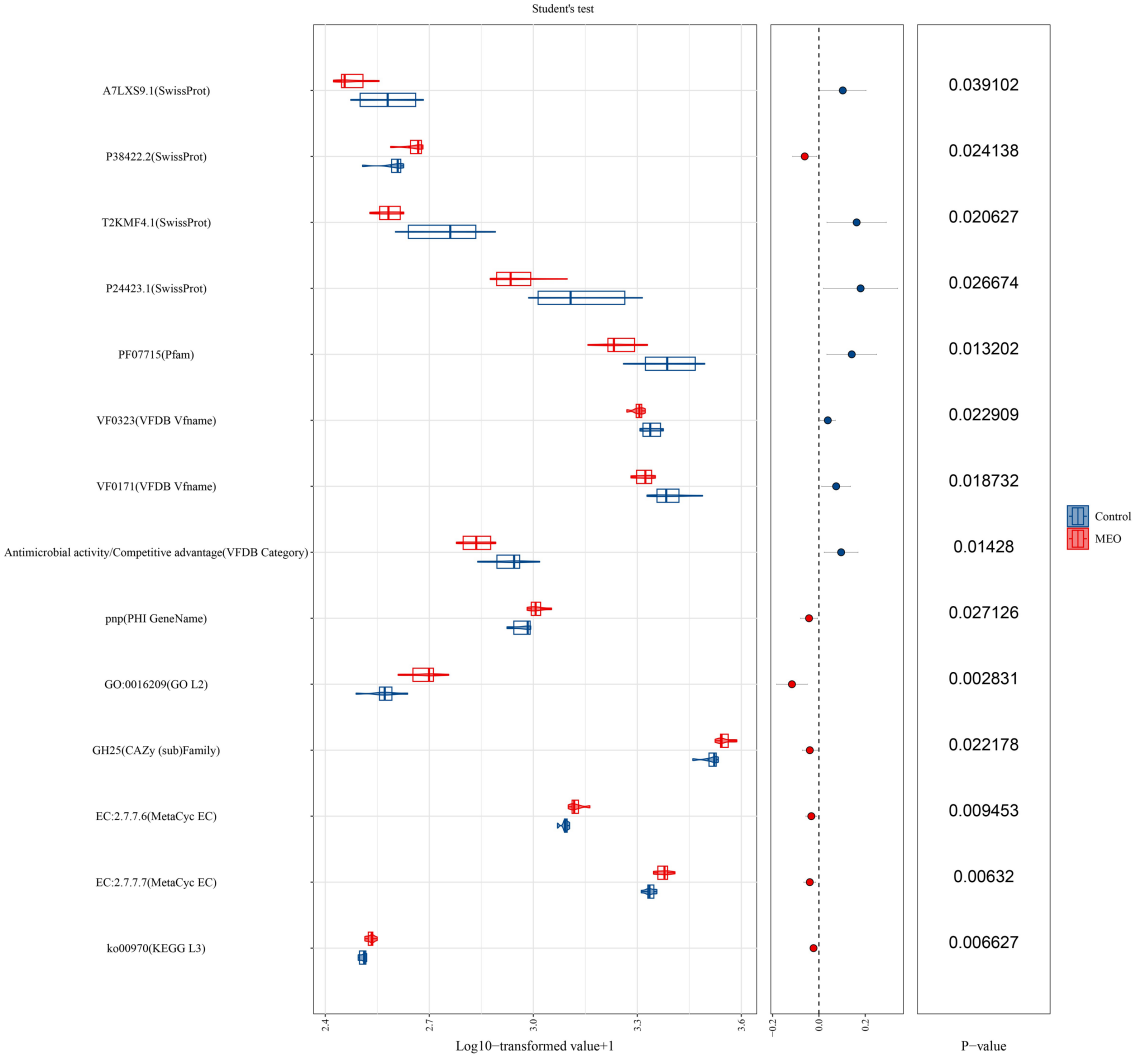
Effects of MEO supplementation on the intestinal microbial functional genes. T (Left) Relative abundance of functional genes quantified as Log_10_ TPM. (Center) Trends of differences. (Right) *p*-values derived from the *t*-test.

We also examined for the presence of functional genes in the microbiomes of our experimental animals. The MEO group (versus control) displayed significant differences in functional genes for DNA expression, virulence factors, antioxidant activity and antimicrobial activity. Differential genes linked to DNA expression were *ko00970* (aminoacyl-tRNA biosynthesis), DNA-directed DNA polymerase, DNA-directed RNA polymerase. Virulence factor genes included *VF0171* (LPS) and *VF0323* (capsule) and antioxidant genes classified under *GO:0016209* and for *GH25* (lysozyme). Additionally, genes for glycogen biosynthesis (MetaCyc database) were significantly decreased for the EO and Chl groups while the toxin activity gene *GO: 0090729* was significantly decreased in the EO group (Figure 6).

### 3.6 Intestinal metabolomics

#### 3.6.1 Intestinal differential metabolite screening

**Figure 7:**
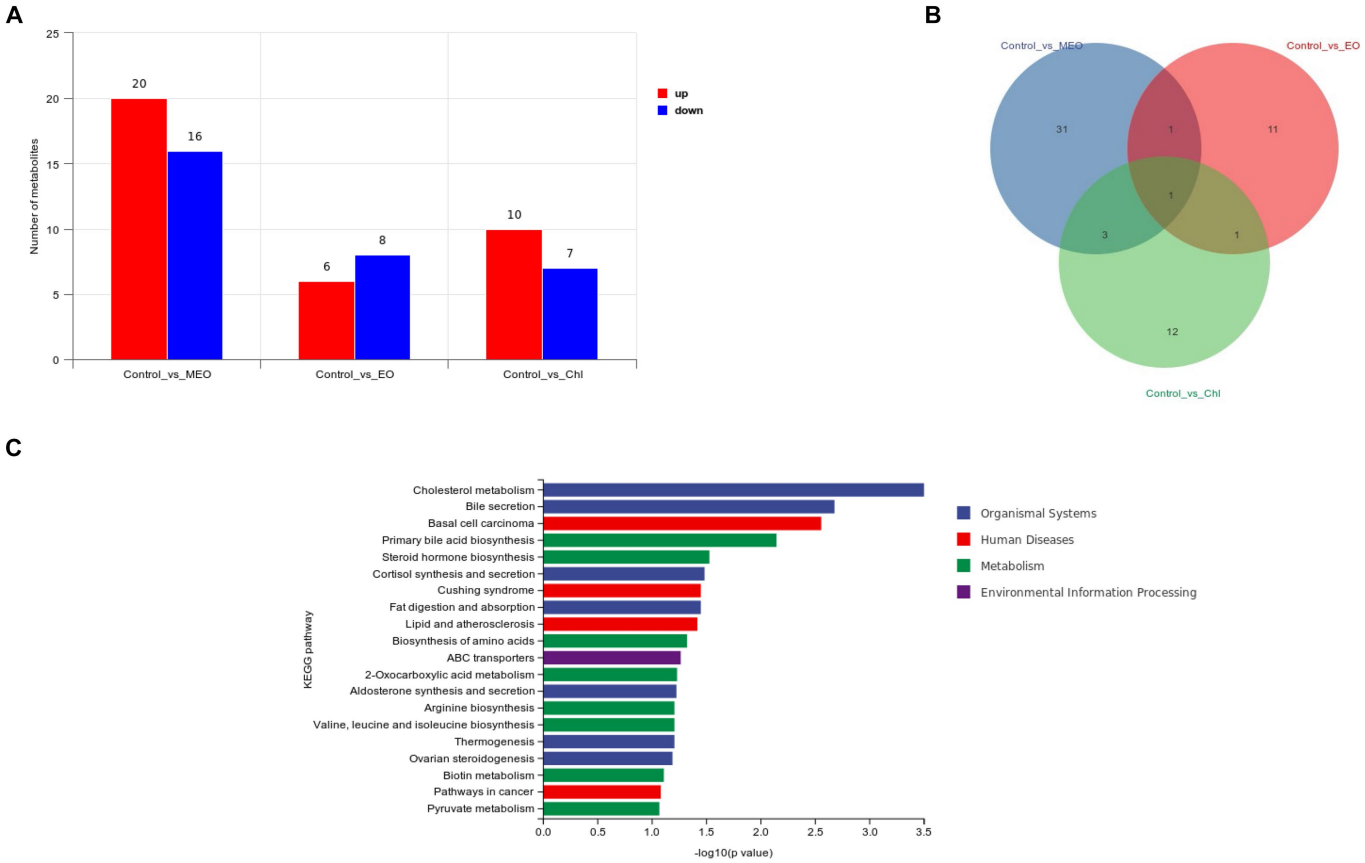
Effects of MEO supplementation on the intestinal microbial metabolites. **(A)** Intergroup differential metabolites. **(B)** Intergroup differential metabolites. **(C)** KEGG enrichment analysis results for differential metabolites.

We performed a metabolomics analysis of our intestinal samples and found 159 significant differential metabolites between control and MEO groups (20 increased and 16 decreased) (Figure 7A). The MEO group also contained 21 unique metabolites (Figure 7B; Supplementary Figures S2–S4). Enrichment analysis of differential substances in the MEO group revealed a number of significant (*p* < 0.05) metabolic pathways including cholesterol metabolism, bile secretion, basal cell carcinoma, primary bile acid biosynthesis, steroid hormone biosynthesis, cortisol synthesis and secretion, Cushing’s syndrome, fat digestion and absorption, lipid and atherosclerosis and biosynthesis of amino acids (Figure 7C).

#### 3.6.2 Intestinal differential metabolites display

Differential metabolites that distinguished the MEO and control groups (VIP-Valve >1 and *p* < 0.05) were also identified. Among these, 5 metabolites related to lipid metabolism included 9,12,13- and 9,10,13- trihydroxy-10-octadecenoic acid, dihydrocortisol, cholesterol and glycochenodeoxycholic acid. We also found 10 metabolites related to amino acid synthesis and metabolism including 2-hydroxycinnamic acid, 3-(4-hydroxyphenyl) pyruvate, gentisic acid, 3,4-dihydroxyhydrocinnamic acid, pipecolic acid, ornithine, tryptophanol, homogentisate, 2-isopropylmalic acid and quinate.

We also identified the lipid metabolites 9,10-epoxyoctadecenoic acid and dicosapentaenoic acid that were significantly increased in the Chl group. Spermidine was significantly increased in the EO group and is linked to the digestive system and amino acid metabolism.

We also identified differential metabolites related to tyrosine metabolism including 3-(4-hydroxyphenyl) pyruvate, gentisic acid and 3,4-dihydroxyhydrocinnamic acid which displayed significant increases in concentration for the MEO group while homogentisate significantly decreased. Differential metabolites related to primary bile acid biosynthesis are cholesterol and glycochenodeoxycholic acid (GCDCA) and both were significantly reduced in concentration in the MEO group (please indicate data location).

### 3.7 Intestinal tetracycline resistance genes

**Figure 8:**
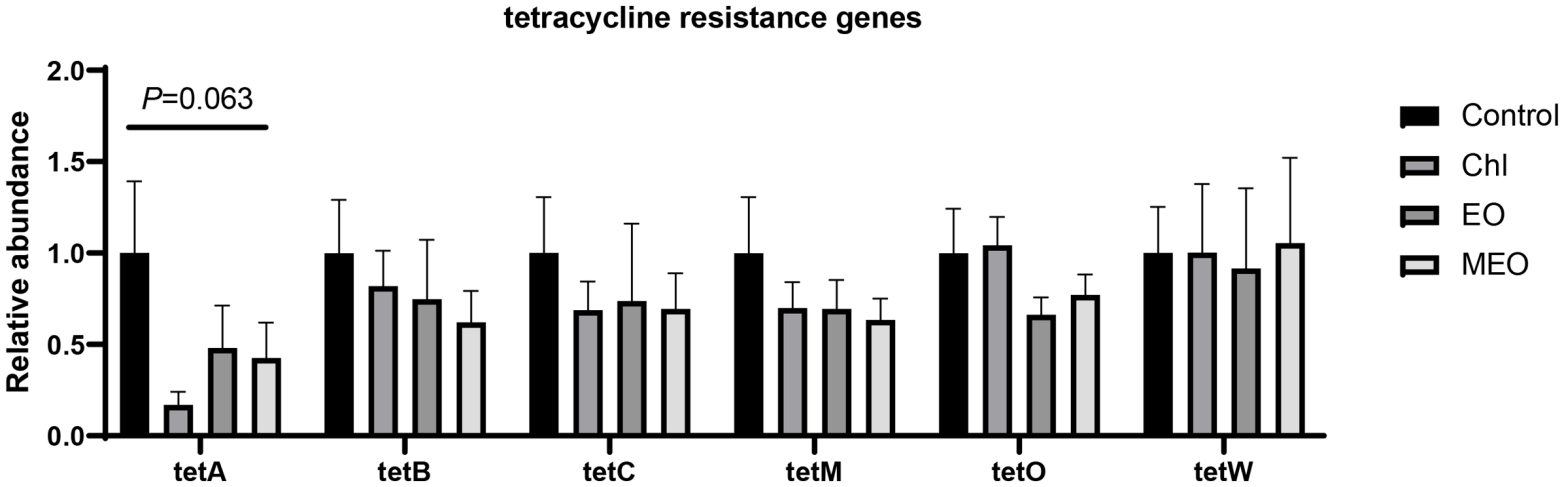
Effects of MEO supplementation on the abundance of intestinal tetracycline resistance genes. Data are represented as means ± SEM, *n* = 12 per group.

In our intestinal samples, we also screened for the presence of tet-resistance genes in *E. coli* that we isolated from plate cultures. We found no significant differences (*p* > 0.05) in the relative content of *tetA*, *tetB*, *tetC*, *tetM*, *tetO* and *tetW* between the four groups including the Chl group (Figure 8).

## 4 Discussion

Previous studies have indicated that thymol, carvacrol and cinnamaldehyde to piglet diets can reduce weaned piglet diarrhea, improve intestinal health, enhance growth and balance the microbiome (28, 29). Microencapsulated essential oils can increase their physical and chemical stability and improve their organoleptic properties while providing controlled/directed release (28, 30). In preliminary studies 100 mg/kg MEO added with one piglet per replicate displayed a significant impact on productivity, intestinal redox indicators, inflammatory biomarkers and intestinal microbiota (18, 31). The current study also found these types of improvements over the control diets and indicated that MEO produced the most significant effect on diarrhea incidence, ADG and F/G than did either the EO and Chl groups. Thus, the current study expanded the findings of the previous studies using increased samples sizes and in a typical farm environment. In addition, we found that MEO significantly increased piglets T-AOC, GSH-Px, jejunal villus height and jejunal VH/CD ratios. A previous study using 4.5% cinnamaldehyde and 13.5% thymol essential oils at 100 mg/kg for 28 days resulted in improved ADG from days 15 to 28 and increased T-AOC, consistent with our results (32). Furthermore, our metagenomic analysis revealed a significantly higher relative abundance of antioxidant activity-related genes (*GO:0016209*) in the MEO group compared to Controls. Thus, increased antioxidant gene mRNA abundance in the colon was positively correlated with the serum test results. These findings underscore a role for MEO in enhancing weaned piglet health, productivity and serum antioxidant capacity.

MEO supplementation also played a positive role in maintaining the intestinal barrier function in the ileum of weaned piglets and reduced inflammation. In particular, expression of biomarker genes indicative of inflammation (*TNF-α*, *TGF-β1* and *IL-6*) were all reduced in the MEO group. *TNF-α* and *TGF-β1* synergistically enhance *IL-6* secretion in intestinal epithelial cells that has a pro-inflammaroty effect (33). *TGF-β1* promotes the production of amphiregulin (AREG) in intestinal epithelial cells that helps strengthen barrier functions and allow tissue repair (34). *TGF-β1* can improve *TNF-α* induced intestinal epithelial barrier disorder by downregulating the expression of the *NOD2* gene in intestinal epithelial cells (34–36). *TNF-α* reduces trans-epithelial electrical resistance and increases the permeability of the intestinal barrier (37), while *IL-6* increases the expression of pro-inflammatory biomarkers (38). These combined processes lead to the disruption of the intestinal epithelial barrier function. Our results indicated that the piglet ileum, MEO reduced *TNF-α* and *IL-6* mRNA levels and increased expression of *TGF-β1* that are all positive influences of the intestinal barrier. Therefore, MEO plays a role in reducing the expression of inflammatory biomarkers to alleviate intestinal inflammatory responses to maintain intestinal barrier integrity.

MEO has also been reported to have positive influences on the intestinal microbiota and also exhibits potent antibacterial activity (39). Yet, supplementation with carvacrol and thymol does not decrease intestinal bacterial abundance in weaned piglets (40). Our results agreed with these findings and MEO did not cause significant differences in the α-diversity of the intestinal microbiome compared to Controls but did reduce the number of unique species and significantly decreased the proportion of the *Bacteroidota* phylum thus increasing the *Firmicutes*/*Bacteroidota* ratio (F/B). In both piglet and human colonic contents, *Firmicutes* and *Bacteroidota* are dominant phyla accounting for up to 90% of the total and have important roles in the intestinal microbiome (41). In our experiments, the proportion in each group reached 86.67–89.53%.

The presence of *Firmicutes* can alter bile acid metabolism and their depletion in IBD patients can elevate primary and conjugated bile acids that potentially impair intestinal barrier function and immunity and thus promote disease progression (42). In our experiments, MEO lowered cholesterol and primary bile acid concentrations suggesting enhanced colonic *Firmicutes* abundance, bile acid metabolism and improved fat absorption in weaned piglets. Additionally, the MEO group exhibited a significant reduction in the Bacteroidota compared to the Control group. We found d reduced gene abundance for *VF0171* (LPS) and *VF0323* (capsule) biosynthesis that play roles in IBD pathogenesis (43) and evading and manipulating the host immune system (44), respectively. LPS administration to piglets could be countered by supplementation of cinnamaldehyde, carvacrol and thymol (45) and might be the result of specific inhibition of Gram-negative bacteria (46).

**Figure 9:**
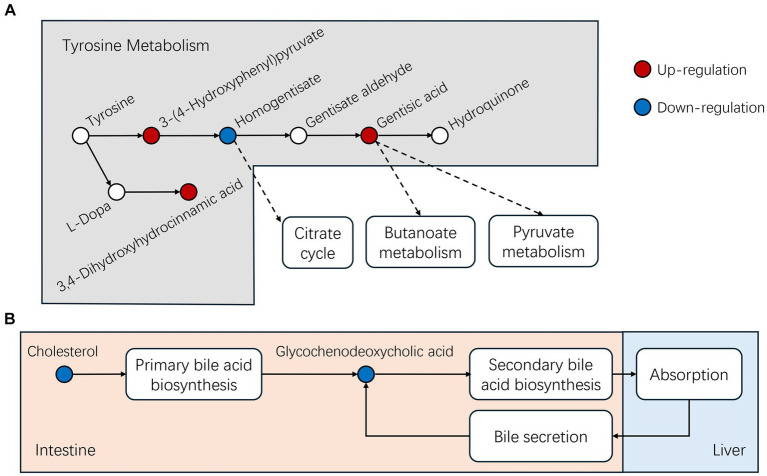
Differential metabolites identified in this study for the following pathways **(A)** Tyrosine and **(B)** bile acid metabolism.

The presence of MEO also influenced tyrosine metabolism via increasing the intermediates 3-(4-Hydroxyphenyl) pyruvate, gentisic acid and 3,4-d dihydroxyhydrocinnamic acid while decreasing homogentisate. Gentisic acid will be continue metabolized via butanoate and pyruvate metabolism or direct conversion to hydroquinone (Figures 9A; Supplementary Figure S6). Butanoate metabolism plays a critical role in maintaining a healthy intestinal environment, influencing intestinal stem cell function and epithelial homeostasis and alteration in metabolic signaling during aging can affect organism resilience and lifespan (47, 48).

MEO also influences bile acid metabolism and we found significant reductions in cholesterol and its metabolic by-product glycochenodeoxycholic acid (GCDCA) levels in the MEO group. Cholesterol metabolizes into GCDCA in the primary bile acid biosynthesis process within intestinal microbiota and GCDCA serves as an intestinal lipid carrier that is transported to the liver where it is absorbed as dietary fat. Simultaneously, the liver also secretes GCDCA into the intestine through bile, thus recycling and absorbing lipids from the intestine (Figure 9B; Supplementary Figures S7, S8). Alterations in the abundance of Bacteroidota and Firmicutes (increased F/B ratio) contributes to a significant decrease in GCDCA present in the intestine (49) These findings suggest that MEO could enhance microbial cholesterol metabolism and improve intestinal fat absorption. In agreement with this, we found that the MEO group displayed increases in linoleic acid metabolism and elevated end products including 9,12,13- and 9,10,13- trihydroxy-10-octadecenoic acid. This metabolism typically takes place in eosinophils in pigs and contributes to skin barrier health but also has implications in colorectal cancer diagnostics and therapy (50, 51).

Previous studies have consistently identified tetracycline resistance genes in pig farming environments, pig-associated *E. coli* and pork products (52, 53). Our metagenomic analysis indicated no significant changes in tetracycline resistance genes in any of our experimental groups including Chl. These results were further validated using qPCR assays. The lack of significant differences in resistant genes may be due to the short-term exposure of Chl group piglets to sub-therapeutic levels (54, 55). Similarly, we demonstrated that the addition of MEO to the diet also did not negatively affect the relative abundance of intestinal *E. coli* resistance genes in weaned piglets.

Current research on the intestinal microbiota of weaned piglets is still limited but metagenomics provides a powerful tool for the direct identification of microbial DNA from samples without the need for microbial culture. When coupled with metabolomics, this type of analysis allows for a more comprehensive understanding of the functions and activities of the intestinal microbiota of weaned piglets under different physiological conditions (56).

## 5 Conclusion

The current study demonstrates that MEO feed supplements given to weaned piglets in an intensive rearing environment reduces the incidence of diarrhea, improves productive performance, enhances antioxidant capacity and lowers ileal inflammatory responses while enhancing the immune barrier of the jejunum. The MEO piglets displayed the most significant effects in reducing diarrhea and increasing the average daily weight gain and feed conversion ratio. MEO most likely improves piglet productive performance in the colon by reducing the proportion of Bacteroidetes, promoting microbial cholesterol and tyrosine metabolism and enhancing intestinal lipid and amino acid utilization.

## Data availability statement

The authors confirm that the data supporting the findings of this study are available within the article or its Supplementary material.

## Ethics statement

The animal study was approved by Animal Ethics Committee of the Experimental Animal Center of South China Agricultural University. The study was conducted in accordance with the local legislation and institutional requirements.

## Author contributions

XX: Data curation, Writing – original draft, Writing – review & editing. KM: Methodology, Writing – original draft, Writing – review & editing. CC: Conceptualization, Data curation, Writing – review & editing. YL: Formal analysis, Software, Writing – review & editing. LgL: Methodology, Software, Writing – review & editing. JX: Formal analysis, Methodology, Writing – review & editing. LiL: Data curation, Formal analysis, Funding acquisition, Writing – review & editing. XH: Data curation, Funding acquisition, Methodology, Writing – original draft, Writing – review & editing.

## References

[1] Tang Q Lan T Zhou C Gao J Wu L Wei H. Nutrition strategies to control post-weaning diarrhea of piglets: from the perspective of feeds. Anim Nutr. (2024). doi: 10.1016/j.aninu.2024.03.006 PMC11127239 38800731

[2] Tang X Xiong K Fang R Li M. Weaning stress and intestinal health of piglets: a review. Front Immunol. (2022) 13:1042778. doi: 10.3389/fimmu.2022.1042778, PMID: 36505434 PMC9730250

[3] Schröder B Hoppe S Breves G. Evidence for down-regulation of neurogenic secretion in small intestinal epithelium from weaned piglets suffering from diarrhea. Livest Sci. (2010) 133:218–21. doi: 10.1016/j.livsci.2010.06.068

[4] Smith F Clark JE Overman BL Tozel CC Huang JH Rivier JEF. Early weaning stress impairs development of mucosal barrier function in the porcine intestine. Am J Physiol Gastrointest Liver Physiol. (2010) 298:G352–63. doi: 10.1152/ajpgi.00081.2009, PMID: 19926814 PMC2838512

[5] Xia B Zhong R Wu W Luo C Meng Q Gao Q. Mucin o-glycan-microbiota axis orchestrates gut homeostasis in a diarrheal pig model. Microbiome. (2022) 10:139. doi: 10.1186/s40168-022-01326-8, PMID: 36045454 PMC9429786

[6] Moeser AJ Ryan KA Nighot PK Blikslager AT. Gastrointestinal dysfunction induced by early weaning is attenuated by delayed weaning and mast cell blockade in pigs. Am J Physiol Gastrointest Liver Physiol. (2007) 293:G413–21. doi: 10.1152/ajpgi.00304.2006, PMID: 17525151

[7] Belkaid Y Hand TW. Role of the microbiota in immunity and inflammation. Cell. (2014) 157:121–41. doi: 10.1016/j.cell.2014.03.011, PMID: 24679531 PMC4056765

[8] Vangroenweghe FA Van Poucke A Defoort P. 236 performance and antibiotic use of piglets vaccinated with an *E. coli* F4/F18 vaccination for the prevention of F18-etec post-weaning diarrhea. J Anim Sci. (2021) 99:138. doi: 10.1093/jas/skab054.232

[9] Yang Q Huang X Wang P Yan Z Sun W Zhao S. Longitudinal development of the gut microbiota in healthy and diarrheic piglets induced by age-related dietary changes. Microbiology. (2019) 8:e923. doi: 10.1002/mbo3.923, PMID: 31496126 PMC6925166

[10] Oropeza-Moe M Grøntvedt CA Phythian CJ Sørum H Fauske AK Framstad T. Zinc oxide enriched peat influence *Escherichia coli* infection related diarrhea, growth rates, serum and tissue zinc levels in Norwegian piglets around weaning: five case herd trials. Porcine Health Manag. (2017) 3:14. doi: 10.1186/s40813-017-0060-7, PMID: 28680702 PMC5488422

[11] Li Y Cao H Zhang S Guo P Zhao J Zhang D. Effects of the supplementation of essential oil mixtures on growth performance, nutrient digestibility, immune status and microbial community in weaned piglets. Animals. (2023) 13:3697. doi: 10.3390/ani13233697, PMID: 38067048 PMC10705780

[12] Canal B Puyalto M Mesas L Sol C Chaves R Cantarelli V. PSIX-17 effect of imprinting with essential oils on performance parameters and diarrheas in weaned piglets. J Anim Sci. (2019) 97:343–4. doi: 10.1093/jas/skz258.685

[13] Xu Y Lahaye L He Z Zhang J Yang C Piao X. Micro-encapsulated essential oils and organic acids combination improves intestinal barrier function, inflammatory responses and microbiota of weaned piglets challenged with enterotoxigenic *Escherichia coli* F4 (K88^+^). Anim Nutr. (2020) 6:269–77. doi: 10.1016/j.aninu.2020.04.004, PMID: 33005760 PMC7503083

[14] Xu Y Lahaye L He Z Zhang J Yang C Piao X. Effects of micro-encapsulated formula of organic acids and essential oils on performance and gut integrity of weaned piglets challenged with ETEC K88. J Anim Sci. (2019) 97:77–8. doi: 10.1093/jas/skz122.142

[15] Jiang XR Awati A Agazzi A Vitari F Ferrari A Bento H. Effects of a blend of essential oils and an enzyme combination on nutrient digestibility, ileum histology and expression of inflammatory mediators in weaned piglets. Animal. (2015) 9:417–26. doi: 10.1017/S1751731114002444 25275341

[16] Granata G Riccobene C Napoli E Geraci C. Polymeric nanocapsules containing fennel essential oil: their preparation, physicochemical characterization, stability over time and in simulated gastrointestinal conditions. Pharmaceutics. (2022) 14:873. doi: 10.3390/pharmaceutics14040873, PMID: 35456707 PMC9026405

[17] Yang C Zhang L Cao G Feng J Yue M Xu Y. Effects of dietary supplementation with essential oils and organic acids on the growth performance, immune system, fecal volatile fatty acids, and microflora community in weaned piglets. J Anim Sci. (2020) 98:skz039. doi: 10.1093/jas/skz039, PMID: 30780162 PMC6979002

[18] Mo K Yu W Li J Zhang Y Xu Y Huang X. Dietary supplementation with a microencapsulated complex ofthymol, carvacrol, andcinnamaldehyde improves intestinal barrier function in weaning piglets. J Sci Food Agric. (2023) 103:1994–2003. doi: 10.1002/jsfa.12322, PMID: 36347590

[19] National Research Council. Nutrient requirements of swin. 11th ed. Washington, DC: The National Academies Press (2012). 420 p.

[20] Yuan L Kang SY Ward LA To TL Saif LJ. Antibody-secreting cell responses and protective immunity assessed in gnotobiotic pigs inoculated orally or intramuscularly with inactivated human rotavirus. J Virol. (1998) 72:330–8. doi: 10.1128/JVI.72.1.330-338.1998, PMID: 9420231 PMC109380

[21] Li M Wang S Li Y Zhao M Kuang J Liang D. Gut microbiota-bile acid crosstalk contributes to the rebound weight gain after calorie restriction in mice. Nat Commun. (2022) 13:2060. doi: 10.1038/s41467-022-29589-7 35440584 PMC9018700

[22] Qiao S Liu C Sun L Wang T Dai H Wang K. Gut *Parabacteroides merdae* protects against cardiovascular damage by enhancing branched-chain amino acid catabolism. Nat Metab. (2022) 4:1271–86. doi: 10.1038/s42255-022-00649-y, PMID: 36253620

[23] Bolyen E Rideout JR Dillon MR Bokulich NA Abnet CC Al-Ghalith GA. Reproducible, interactive, scalable and extensible microbiome data science using QIIME 2. Nat Biotechnol. (2019) 37:852–7. doi: 10.1038/s41587-019-0209-9, PMID: 31341288 PMC7015180

[24] Segata N Izard J Waldron L Gevers D Miropolsky L Garrett WS. Metagenomic biomarker discovery and explanation. Genome Biol. (2011) 12:R60. doi: 10.1186/gb-2011-12-6-r60, PMID: 21702898 PMC3218848

[25] Turroni S Fiori J Rampelli S Schnorr SL Consolandi C Barone M. Fecal metabolome of the hadza hunter-gatherers: a host-microbiome integrative view. Sci Rep. (2016) 6:32826. doi: 10.1038/srep32826, PMID: 27624970 PMC5021991

[26] Zelena E Dunn WB Broadhurst D Francis-Mcintyre S Carroll KM Begley P. Development of a robust and repeatable UPLC-MS method for the long-term metabolomic study of human serum. Anal Chem. (2009) 81:1357–64. doi: 10.1021/ac8019366, PMID: 19170513

[27] Want EJ Masson P Michopoulos F Wilson ID Theodoridis G Plumb RS. Global metabolic profiling of animal and human tissues via UPLC-MS. Nat Protoc. (2013) 8:17–32. doi: 10.1038/nprot.2012.135, PMID: 23222455

[28] Partheniadis I Koukourikou M Tsalavouti D Nikolakakis I. Preparation, characterization, and in vitro release of microencapsulated essential oil hydroxyapatite pellets filled into multifunctional capsules. J Drug Deliv Sci Technol. (2023) 80:104114. doi: 10.1016/j.jddst.2022.104114

[29] Rebucci R Staurenghi V Marchetti L Giromini C Bontempo V. Effects of nature identical essential oils (carvacrol, thymol and cinnamaldehyde) on growth performance of piglets and non-invasive markers of antioxidant status and calprotectin release. Livest Sci. (2022) 263:104959. doi: 10.1016/j.livsci.2022.104959

[30] Da Veiga RDS Da Silva-Buzanello RA Corso MP Canan C. Essential oils microencapsulated obtained by spray drying: a review. J Essent Oil Res. (2019) 31:457–73. doi: 10.1080/10412905.2019.1612788

[31] Mo K Li J Liu F Xu Y Huang X Ni H. Superiority of microencapsulated essential oils compared with common essential oils and antibiotics: effects on the intestinal health and gut microbiota of weaning piglet. Front Nutr. (2022) 8:808106. doi: 10.3389/fnut.2021.808106, PMID: 35096948 PMC8790512

[32] Tian Q Piao X. Essential oil blend could decrease diarrhea prevalence by improving antioxidative capability for weaned pigs. Animals. (2019) 9:847. doi: 10.3390/ani9100847, PMID: 31640257 PMC6826739

[33] Mcgee DW Bamberg T Vitkus SJ Mcghee JR. A synergistic relationship between TNF-alpha, IL-1 beta, and TGF-beta 1 on IL-6 secretion by the IEC-6 intestinal epithelial cell line. Immunology. (1995) 86:6–11. PMID: 7590883 PMC1383803

[34] Chen F Yang W Huang X Cao AT Bilotta AJ Xiao Y. Neutrophils promote amphiregulin production in intestinal epithelial cells through TGF-β and contribute to intestinal homeostasis. J Immunol. (2018) 201:2492–501. doi: 10.4049/jimmunol.1800003, PMID: 30171165 PMC6179911

[35] Rosenstiel P Fantini M Bräutigam K Kühbacher T Waetzig GH Seegert D. TNF-alpha and IFN-gamma regulate the expression of the NOD2 (CARD15) gene in human intestinal epithelial cells. Gastroenterology. (2003) 124:1001–9. doi: 10.1053/gast.2003.50157, PMID: 12671897

[36] Xiao K Cao S Jiao L Song Z Lu J Hu C. TGF-β1 protects intestinal integrity and influences Smads and MAPK signal pathways in IPEC-J2 after TNF-α challenge. Innate Immun. (2017) 23:276–84. doi: 10.1177/1753425917690815 28142299

[37] Droessler L Cornelius V Markov AG Amasheh S. Tumor necrosis factor alpha effects on the porcine intestinal epithelial barrier include enhanced expression of TNF receptor 1. Int J Mol Sci. (2021) 22:8746. doi: 10.3390/ijms22168746, PMID: 34445450 PMC8395858

[38] Pan Z Huang J Hu T Zhang Y Zhang L Zhang J. Protective effects of selenium nanoparticles against bisphenol A-induced toxicity in porcine intestinal epithelial cells. Int J Mol Sci. (2023) 24:7242. doi: 10.3390/ijms24087242, PMID: 37108405 PMC10139072

[39] García-Salinas S Elizondo-Castillo H Arruebo M Mendoza G Irusta S. Evaluation of the antimicrobial activity and cytotoxicity of different components of natural origin present in essential oils. Molecules. (2018) 23:1399. doi: 10.3390/molecules23061399, PMID: 29890713 PMC6100501

[40] Michiels J Missotten J Van Hoorick A Ovyn A Fremaut D De Smet S. Effects of dose and formulation of carvacrol and thymol on bacteria and some functional traits of the gut in piglets after weaning. Arch Anim Nutr. (2010) 64:136–54. doi: 10.1080/17450390903499915, PMID: 20481352

[41] Rinninella E Raoul P Cintoni M Franceschi F Miggiano G Gasbarrini A. What is the healthy gut microbiota composition? A changing ecosystem across age, environment, diet, and diseases. Microorganisms. (2019) 7:14. doi: 10.3390/microorganisms7010014 30634578 PMC6351938

[42] Andoh A Nishida A. Alteration of the gut microbiome in inflammatory bowel disease. Digestion. (2023) 104:16–23. doi: 10.1159/000525925 35901721

[43] Kim S Shin M Kim S. Lipocalin 2 activates the NLRP3 inflammasome via LPS‑induced NF‑κB signaling and plays a role as a pro‑inflammatory regulator in murine macrophages. Mol Med Rep. (2022) 26:358. doi: 10.3892/mmr.2022.12875, PMID: 36263611 PMC9608086

[44] Brock SR Parmely MJ. *Francisella tularensis* confronts the complement system. Front Cell Infect Microbiol. (2017) 7:523. doi: 10.3389/fcimb.2017.00523, PMID: 29312899 PMC5742141

[45] Zhang Y Li Q Wang Z Dong Y Yi D Wu T. Dietary supplementation with a complex of cinnamaldehyde, carvacrol, and thymol negatively affects the intestinal function in lps-challenged piglets. Front Vet Sci. (2023) 10:1098579. doi: 10.3389/fvets.2023.1098579 37065240 PMC10097997

[46] Helander IM Alakomi H Latva-Kala K Mattila-Sandholm T Pol I Smid EJ. Characterization of the action of selected essential oil components on gram-negative bacteria. J Agric Food Chem. (1998) 46:3590–5. doi: 10.1021/jf980154m

[47] Funk MC Zhou J Boutros M. Ageing, metabolism and the intestine. EMBO Rep. (2020) 21:e50047. doi: 10.15252/embr.202050047, PMID: 32567155 PMC7332987

[48] Louis P Flint HJ. Diversity, metabolism and microbial ecology of butyrate-producing bacteria from the human large intestine. FEMS Microbiol Lett. (2009) 294:1–8. doi: 10.1111/j.1574-6968.2009.01514.x, PMID: 19222573

[49] Pardesi B Roberton AM Lee KC Angert ER Rosendale DI Boycheva S. Distinct microbiota composition and fermentation products indicate functional compartmentalization in the hindgut of a marine herbivorous fish. Mol Ecol. (2022) 31:2494–509. doi: 10.1111/mec.16394, PMID: 35152505 PMC9306998

[50] Barry EL Fedirko V Jin Y Liu K Mott LA Peacock JL. Plasma metabolomics analysis of aspirin treatment and risk of colorectal adenomas. Cancer Prev Res. (2022) 15:521–31. doi: 10.1158/1940-6207.CAPR-21-0555, PMID: 35653338 PMC9357068

[51] Vidman L Zheng R Bodén S Ribbenstedt A Gunter MJ Palmqvist R. Untargeted plasma metabolomics and risk of colorectal cancer—an analysis nested within a large-scale prospective cohort. Cancer Metab. (2023) 11:17. doi: 10.1186/s40170-023-00319-x, PMID: 37849011 PMC10583301

[52] Kallau N Wibawan I Lukman D Sudarwanto M. Detection of multi-drug resistant (mdr) *Escherichia coli* and *tet* gene prevalence at a pig farm in Kupang, Indonesia. J Adv Vet Anim Res. (2018) 5:388–96. doi: 10.5455/javar.2018.e289, PMID: 31453148 PMC6702907

[53] Liu Z Klümper U Shi L Ye L Li M. From pig breeding environment to subsequently produced pork: comparative analysis of antibiotic resistance genes and bacterial community composition. Front Microbiol. (2019) 10:43. doi: 10.3389/fmicb.2019.00043 30761096 PMC6361818

[54] Baskerville K Funk J. (2005). Effect of chlortetracycline on the distribution of resistance genes. Iowa State University Conferences and Symposia

[55] Stabler SL Fagerberg DJ Quarles CL. Effects of oral and injectable tetracyclines on bacterial drug resistance in feedlot cattle. Am J Vet Res. (1982) 43:1763–6. 6924552

[56] Wani AK Hashem NM Akhtar N Singh R Madkour M Prakash A. Understanding microbial networks of farm animals through genomics, metagenomics and other meta-omic approaches for livestock wellness and sustainability—a review. Ann Anim Sci. (2022) 22:839–53. doi: 10.2478/aoas-2022-0002

