# Supplementary material for: Microencapsulated essential oils alleviate diarrhea in weaned piglets by modulating the intestinal microbial barrier as well as not inducing antibiotic resistance: a field research
Source: Front Vet Sci. 2024 May 10;11:1396051. doi: 10.3389/fvets.2024.1396051 (PMC11117338; doi:10.3389/fvets.2024.1396051)
Supplement: Supplementary file 1 [file Data_Sheet_1.docx]

**Supplementary Material**

1. **Supplementary Tables**

**Table S1.** Primer sequences for target genes

| Gene | Sequence of primers (5′ to 3′) | GenBank accession No. |
| --- | --- | --- |
| *IL-1β* | F: GAAAGATAACACGCCCACCC | NM_214055.1 |
|  | R: TCTGCTTGAGAGGTGCTGATGT |  |
| *IL-6* | F: CCTGTCCACTGGGCACATAAC | NM_214399.1 |
|  | R: CAAGAAACACCTGGCTCTGAAAC |  |
| *IL-10* | F: CAAGGAGTTGTTTCCGTTA | NM_010548.2 |
|  | R: GCCATGAATGAATTTGACA |  |
| *TNF-a* | F: CATCGCCGTCTCCTACCA | NM_214022.1 |
|  | R: CCCAGATTCAGCAAAGTCCA |  |
| *IFN -γ* | F: GAGCCAAATTGTCTCCTTCTAC | NM_213948.1 |
|  | R: CGAAGTCATTCAGTTTCCCAG |  |
| *TGF-β1* | F: GGACCTTATCCTGAATGCCTT | NM_214015.1 |
|  | R: TAGGTTACCACTGAGCCACAAT |  |
| *ZO-1* | F: CAGAGACCAAGAGCCGTCC | XM_021098827.1 |
|  | R: TGCTTCAAGACATGGTTGGC |  |
| *Occludin* | F: TCAGGTGCACCCTCCAGATT | NM_001163647.2 |
|  | R: AGGAGGTGGACTTTCAAGAGG |  |
| *Claudin-1* | F: AAACCGTGTGGGAACAACCA | NM_001244539.1 |
|  | R: TTTAAGGACCGCCCTCTCCC |  |
| *GAPDH* | F: ATGGTGAAGGTCGGAGTGAAC | NM_001206359.1 |
|  | R: CTCGCTCCTGGAAGATGGT |  |

*IL-1β* = Interleukin-1β; *IL-6* = Interleukin-6; *IL-10* = Interleukin-10; *TNF-a* = Tumor Necrosis Factor-α; *IFN -γ=* Interferon-γ; *ZO-1* = Zonula occludens-1; *GAPDH* = Glyceraldehyde-3-phosphate dehydrogenase.

**Table S2.** Primer sequences for tetracycline resistance genes

| Gene | Sequence of primers (5′ to 3′) | GenBank accession No. |
| --- | --- | --- |
| tetA | F: GCTACATCCTGCTTGCCTTC | X61367 |
|  | R: CATAGATCGCCGTGAAGAGG |  |
| tetB | F: GCCCAGTGCTGTTGTTGTCAT | J01830 |
|  | R: CGTTTTTTCGCCCCATTTAGT |  |
| tetC | F: CTTGAGAGCCTTCAACCCAG | J01749 |
|  | R: ATGGTCGTCATCTACCTGCC |  |
| tetM | F: GTGGACAAAGGTACAACGAG | X90939 |
|  | R: CGGTAAAGTTCGTCACACAC |  |
| tetO | F: ACGGARAGTTTATTGTATACC | Y07780 |
|  | R: TGGCGTATCTATAATGTTGAC |  |
| tetW | F: GAGAGCCTGCTATATGCCAGC | M37031 |
|  | R: GGGCGTATCCACAATGTTAAC |  |
| 16S rDNA | F: CCTACGGGAGGCAGCAG | J01859 |
|  | R: TTACCGCGGCTGCTGGCAC |  |

1. **Supplementary Figures**


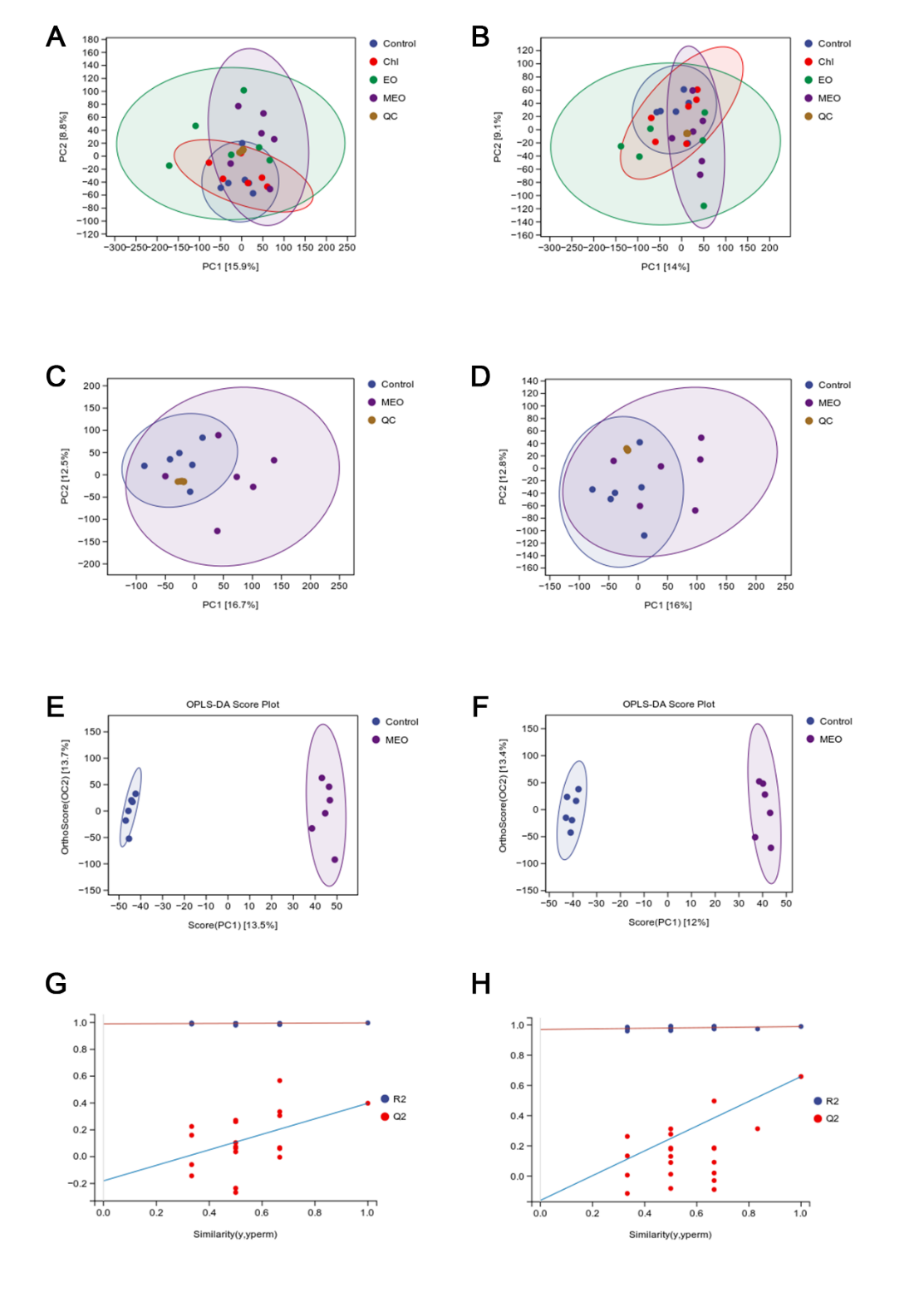


**Figure S1.** Result of Differential Metabolite Screening. (A, B)PCA score plots based on the colonic metabolic profiles of all 4 groups in the positive and negative modes, with R2X values 0.513(positive) and 0.546(negative); (C, D)PCA score plots based on the colonic metabolic profiles of Control and MEO groups in the positive and negative modes, with R2X values 0.531(positive) and 0.517(negative); (E, F)OPLS-DA score plots of Control and MEO group in the positive and negative modes; (G, H)permutation tests of the OPLS-DA model, OPLS-DA Positive mode: R2X = 0.271, R2Y = 0.997, Q2 = 0.398; Negative mode: R2X = 0.304, R2Y = 0.990, Q2 = 0.659.


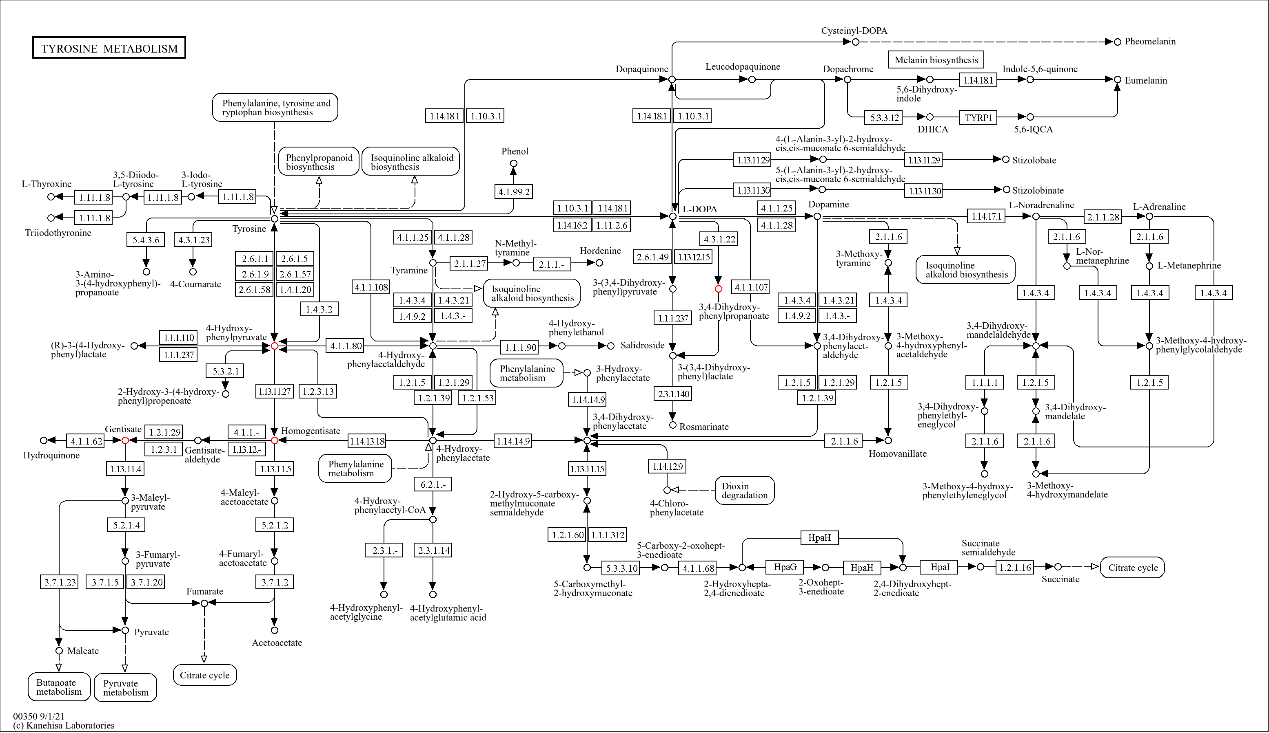


**Figure S2.**  Diagram of differential metabolites in tyrosine metabolism pathway, generated using the KEGG PATHWAY Database (<https://www.genome.jp/kegg/pathway.html>). Differential metabolites are marked with red circles. Reproduced with permission.


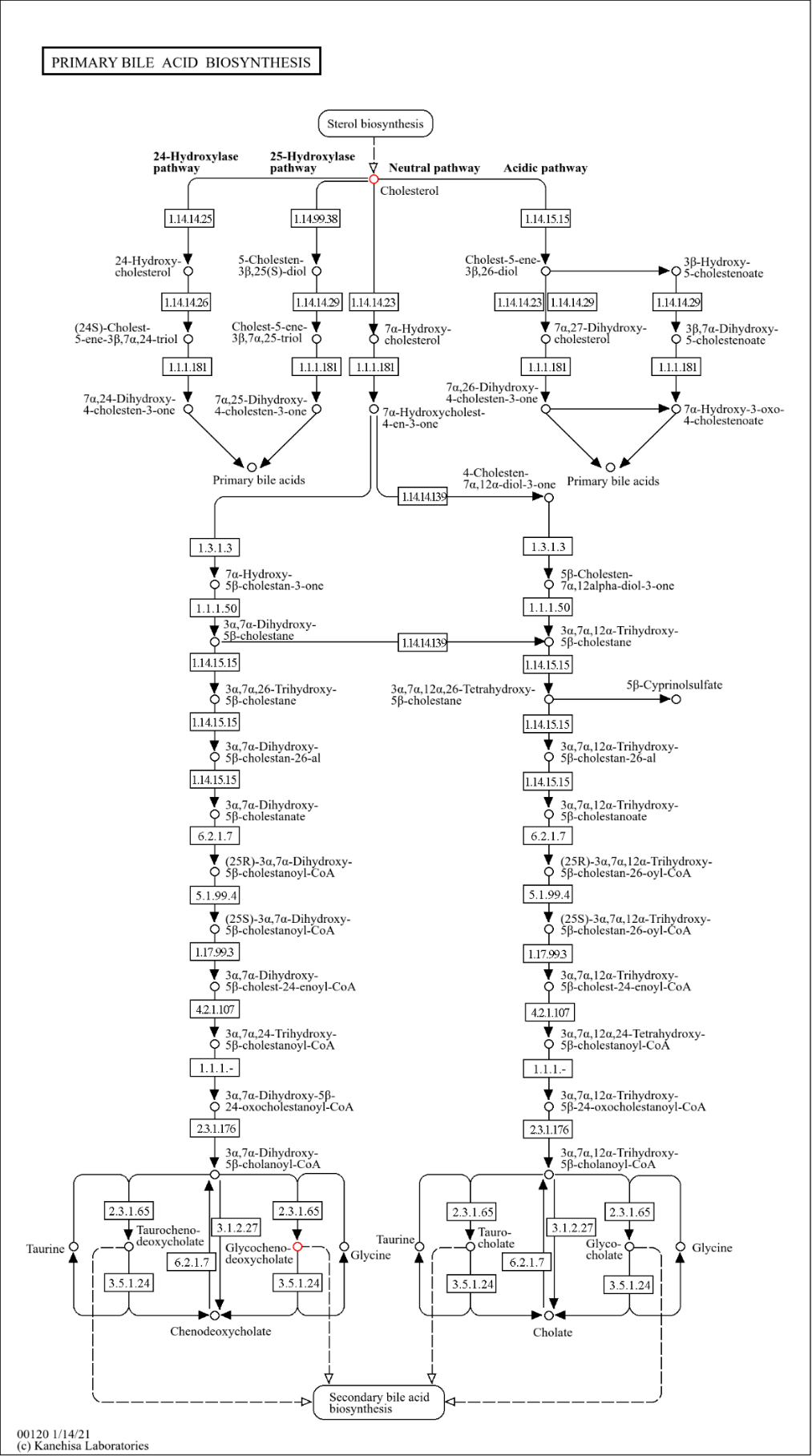


**Fig S3.**  Diagram of differential metabolites in Primary bile acid biosynthesis, generated using the KEGG PATHWAY Database (<https://www.genome.jp/kegg/pathway.html>). Differential metabolites are marked with red circles. Reproduced with permission.


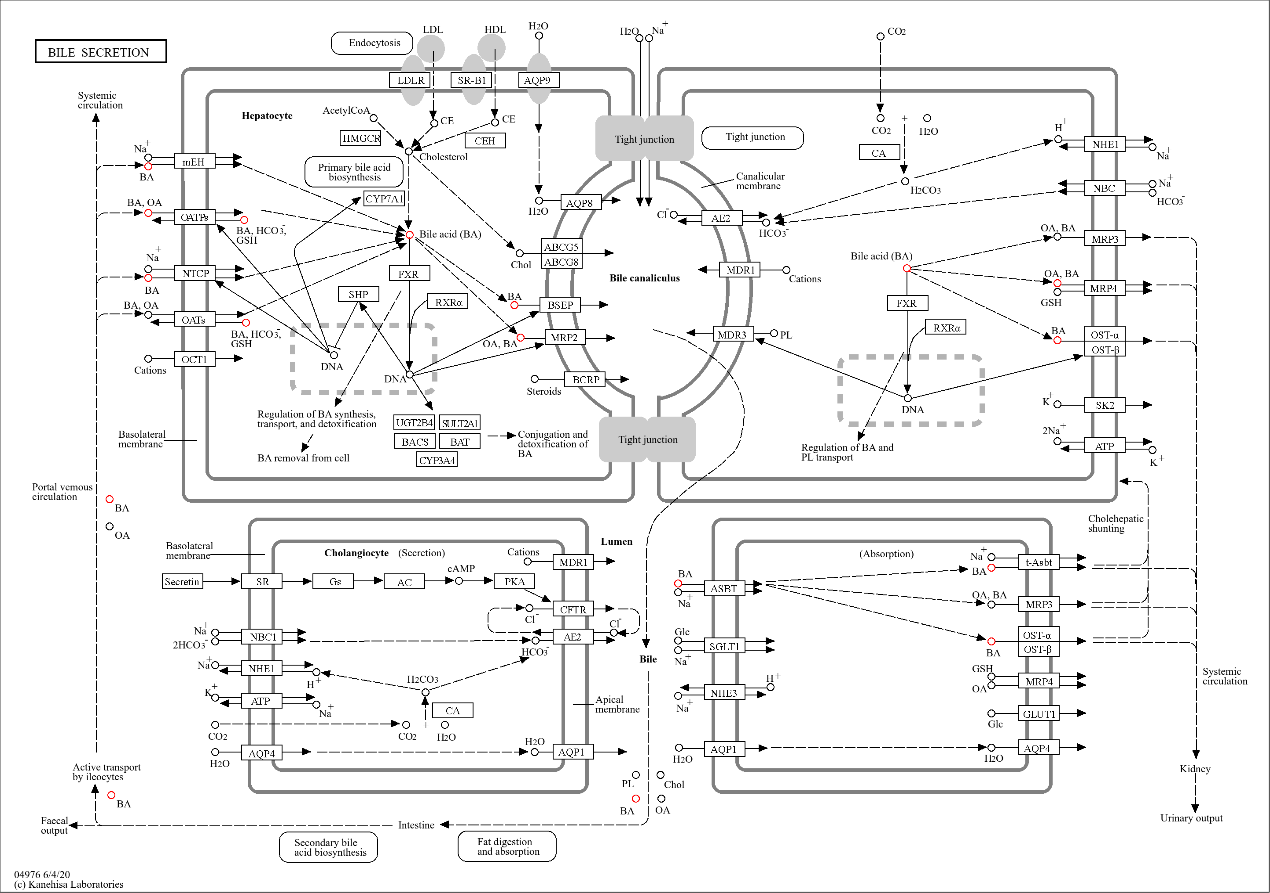


**Figure S4.**  Diagram of differential metabolites in bile secretion, generated using the KEGG PATHWAY Database (<https://www.genome.jp/kegg/pathway.html>). Differential metabolites are marked with red circles. Reproduced with permission.
